# Optimization of Neurite Tracing and Further Characterization of Human Monocyte-Derived-Neuronal-like Cells

**DOI:** 10.3390/brainsci11111372

**Published:** 2021-10-20

**Authors:** Alfredo Bellon, Tuna Hasoglu, Mallory Peterson, Katherine Gao, Michael Chen, Elisabeta Blandin, Alonso Cortez-Resendiz, Gary A. Clawson, Liyi Elliot Hong

**Affiliations:** 1 Department of Psychiatry and Behavioral Health, Penn State Hershey Medical Center, Hershey, PA 17033, USA; thasoglu@pennstatehealth.psu.edu (T.H.); acortezresendiz@pennstatehealth.psu.edu (A.C.-R.); 2 Department of Pharmacology, Penn State Hershey Medical Center, Hershey, PA 17033, USA; 3 Department of Engineering Science and Mechanics, Penn State College of Engineering, State College, Philadelphia, PA 19107, USA; mpeterson1@pennstatehealth.psu.edu; 4 Department of Psychiatry & Human Behavior, Thomas Jefferson University, Philadelphia, PA 19107, USA; Katherine.c.gao@gmail.com; 5 Department of Psychiatry, Lehigh Valley Health Network, 2545 Schoenersville Road, Bethlehem, PA 18017, USA; Michael.Chen@lvhn.org; 6 Department of Neural & Behavioral Sciences, Penn State Hershey Medical Center, Hershey, PA 17033, USA; emblandin@yahoo.com; 7 Department of Pathology, Penn State University College of Medicine, Gittlen Cancer Research Laboratories, Hershey, PA 17033, USA; gac4gac4@gmail.com; 8 Department of Psychiatry, Maryland Psychiatric Research Center, University of Maryland School of Medicine, Baltimore, MD 21201, USA; Ehong@som.umaryland.edu

**Keywords:** schizophrenia, autism, stem cells, cytoskeleton, neurite, dendrite, neurodevelopment, biomarker, transdifferentiation and neuronal model

## Abstract

Deficits in neuronal structure are consistently associated with neurodevelopmental illnesses such as autism and schizophrenia. Nonetheless, the inability to access neurons from clinical patients has limited the study of early neurostructural changes directly in patients’ cells. This obstacle has been circumvented by differentiating stem cells into neurons, although the most used methodologies are time consuming. Therefore, we recently developed a relatively rapid (~20 days) protocol for transdifferentiating human circulating monocytes into neuronal-like cells. These monocyte-derived-neuronal-like cells (MDNCs) express several genes and proteins considered neuronal markers, such as MAP-2 and PSD-95. In addition, these cells conduct electrical activity. We have also previously shown that the structure of MDNCs is comparable with that of human developing neurons (HDNs) after 5 days in culture. Moreover, the neurostructure of MDNCs responds similarly to that of HDNs when exposed to colchicine and dopamine. In this manuscript, we expanded our characterization of MDNCs to include the expression of 12 neuronal genes, including tau. Following, we compared three different tracing approaches (two semi-automated and one automated) that enable tracing using photographs of live cells. This comparison is imperative for determining which neurite tracing method is more efficient in extracting neurostructural data from MDNCs and thus allowing researchers to take advantage of the faster yield provided by these neuronal-like cells. Surprisingly, it was one of the semi-automated methods that was the fastest, consisting of tracing only the longest primary and the longest secondary neurite. This tracing technique also detected more structural deficits. The only automated method tested, Volocity, detected MDNCs but failed to trace the entire neuritic length. Other advantages and disadvantages of the three tracing approaches are also presented and discussed.

## 1. Introduction

Neurodevelopmental disorders such as autism and schizophrenia are relatively common alignments [1,2] caused by a complex combination of environmental and genetic factors. Unfortunately, treatment and diagnostic methods for these illnesses remain unsatisfactory. It is therefore not surprising that the search for biomarkers is intense [3,4,5,6,7,8]. However, a crucial step in the development of biomarkers and improvement of treatment as well as diagnosis for any illness is understanding its pathophysiology.

One of the many challenges researchers face when studying neurodevelopmental disorders is that they are diagnosed once most neurodevelopmental stages have been completed. For instance, schizophrenia is diagnosed in late adolescence or early adulthood. Autism is often recognized earlier in life but still too late to study neuronal processes such as neurite formation, neuronal polarization and pruning of neuronal extensions. These neurodevelopmental processes are of particular importance, as the neuronal structure has been consistently associated with the pathophysiology of schizophrenia and autism [9,10,11,12,13]. It is therefore possible that studying early neurostructural rearrangements directly in cells from patients with autism or schizophrenia would lead to a better understanding of its pathophysiology.

An additional challenge when ascertaining neurodevelopmental disorders is the accessibility of neurons coming directly from clinical patients. This obstacle has been circumvented by several different methods. The collection of olfactory neuroepithelial cells (ONCs) is the only approach presently available that provides access to mature neurons [14]. It also delivers glial, epithelial and neuroprogenitor cells as well as neurons at different stages of differentiation [15,16]. In order to access ONCs, a qualified otorhinolaryngologist has to perform a biopsy of the olfactory mucosa [16]. This invasive procedure has limited the use of ONCs. In addition, concerns have been raised about the reproducibility of data when using olfactory mucosa, as biopsies from the same individual can deliver variable results [15,16]. Another approach that circumvents the limited access of neurons coming directly from patients is the use of mesenchymal stem cells (MSCs). MSCs can be rapidly differentiated into neuronal-like cells in vitro [17]. However, the scarce use of MSCs in the study of psychiatric and neurologic disorders appears to be due to difficulties in retrieving MSCs. Obtaining MSCs, often if not always, requires a biopsy [18], which is a surgical procedure that requires consultation with a specialist. There is also another characteristic of MSCs that has determined its fate in research: the fact that MSCs do not trigger an immunological reaction. Such an attribute makes this type of stem cells an excellent tool for cellular transplant [18]. On the other hand, to study neurodevelopmental disorders, the most common stem cells currently used are induced pluripotent stem cells (IPSCs). IPSCs allow researchers to develop different types of neurons with sophisticated neuropils [19]. Even brain organoids that resemble aspects of early brain development can be generated using IPSCs [19]. Unfortunately, generating IPSCs requires altering the cell’s genome (reprograming) [20,21], which can become a confounder when studying illnesses with poorly understood genetic predispositions, such as autism and schizophrenia [22]. IPSCs have also been criticized because of difficulties in reproducibility [23,24]. Moreover, the transformation of somatic cell to differentiated neuron is expensive and time consuming [15]. Not surprisingly, published manuscripts involving IPSCs and neurodevelopmental disorders comprise rather small cohorts. Another emerging methodology consists of directly reprogramming somatic cells, often fibroblasts, directly into neurons [25,26]. This approach, known as induced neurons (iNs), bypasses the need for dedifferentiation but still requires altering the cell’s genome [25,26]. The potential confounding effects of reprograming and the need to show reproducible results when studying neurodevelopmental disorders remain. However, iNs are becoming a promising alternative for regenerative medicine [27]. A faster approach for obtaining neuronal-like cells that completely avoids genetic reprogramming is transdifferentiation of somatic cells.

We have recently developed a methodology for transdifferentiating human circulating monocytes into neuronal-like cells in only 20 days [28]. These monocyte-derived-neuronal-like cells (MDNCs) express several genes and proteins considered neuronal markers. Among the genes and proteins present in MDNCs are NeuN and PSD-95, considered markers for mature neurons. However, MDNCs also express markers of immature neurons such as nestin. Moreover, these cells are not yet committed to developing into any specific neuronal type and instead express markers for glutamatergic, dopaminergic, GABAergic and serotoninergic neurons. Of particular importance for the study of the neuronal structure is the expression of microtubule associated protein 2 (MAP-2) [28], as this protein is a marker for dendrites [29,30]. Tau is another relevant neuronal protein, as it is an axonal marker [29,30]. Immature neuronal extensions that have not yet developed into either axons or dendrites are called neurites [31]. During early stages of neuronal development, MAP-2 is present in all neurites and in the cell soma [29,30]. We have previously shown that MDNCs express MAP-2 in all its extensions as well as in the soma [28]. While expression of tau in MDNCs is still to be proven, the information currently available indicates that MDNCs extend neurites that have not yet developed into either dendrites or axons. However, even at this early stage of neurodevelopment, we have shown that MDNCs conduct electrical activity [28]. 

In a prior publication, we directly compared the structure of MDNCs with that of human developing neurons (after 5 days in culture) as well as with that of differentiated human neuroblastoma cells [28]. The structure of these three different neuronal cell types was similar [28]. Perhaps more important for the study of schizophrenia and autism is that the structure of MDNCs responds similarly to human neurons and neuroblastoma cells when exposed to dopamine and colchicine [28]. Therefore, MDNCs allow us to study some aspects of the neuronal structure that take place during early development directly in patients’ cells that carry the genetic predisposition to illnesses such as schizophrenia and autism. This opens the possibility of starting to unveil the pathophysiology of such neurodevelopmental disorders. While other neurodevelopmental disorders such as attention deficit hyperactivity disorder (ADHD) [32], bipolar disorder [33] and others can also be studied using MDNCs, here we emphasize autism and schizophrenia because deficits in the neuronal structure are consistently found [9,10,11,12,13]. 

Neurite outgrowth is a key neuronal feature, and therefore characterizing neurites is important for understanding MDNCs’ neuronal properties and their application in disease and pharmacology research. Neurites are numerous, and accurate measurements through individual neurite tracing are labor-intensive. Therefore, in order to take advantage of this faster yield of neuronal-like cells, an efficient neurite tracing method is critical for extracting neurostructural data from MDNCs. The current available options can be divided into two general tracing methodologies: automated and semi-automated. Most automated alternatives are similar. They rely on software capable of detecting neurons stained with a fluorochrome. Tagged cells are automatically traced. The output of such softwares is faster than semi-automated methods, as these latter options require the researcher to select the cell to be traced and then identify the beginning and end of the neurite of interest. One of the advantages of semi-automated methods is that researchers have more flexibility when deciding which cells to trace, as they are not bound by the expression of a specific marker. Another advantage is that immunofluorescence or the expression of a fluorescent marker such as green fluorescent protein (GFP) can be avoided. These techniques are not always desirable, as both can lead to cellular damage [34,35].

Postmortem studies indicate that defects in the neuronal structure of patients with neurodevelopmental disorders are subtle [9,10,11,12,13]. Thus, avoiding immunofluorescence or the expression of a fluorescent marker is advantageous, as these techniques could mask inconspicuous defects [34,35]. Another strategy for minimizing structural confounders is using each neuronal-like cell as its own control. For this purpose, cells are identified and photographed before receiving any treatment. Then, after treatment with the compound to be tested and once the desired incubation time has passed, the exact same cells are again identified and photographed. Structural differences in the same cells before and after treatment are reported. We used this approach to determine the structural effects of colchicine and dopamine on MDNCs [28].

In order to avoid the use of fluorochromes and to utilize each neuronal-like cell as its own control, we decided to test three different neurite tracing approaches. First, we traced each MDNC in its entirety using a semi-automated method. Since this approach is lengthy, we also tested a simplified version in which only the longest primary neurite and longest secondary neurite were traced. Finally, the third and most intriguing alternative was to use an automated method without the addition of a fluorochrome. Automated software are triggered by brightness. Neuronal-like cells appear significantly brighter than the background when pictures are taken using light microscopy, and thus, cell recognition is expected.

The first goal of this study was to compare the expression of several previously unreported neuronal markers between MDNCs, human neuroblastoma cells and THP-1 cells (a human monocytic cell line) to further validate MDNCs as neuronal-like cells. The second and main objective was to determine which of the three neurite tracing methods was faster. We hypothesized that an automated method would be faster than the other two semi-automated approaches. The third and final goal was to establish whether any of the three techniques was better at exposing neurostructural defects, with the expectation that whole-cell tracing of MDNCs would reveal the highest number of structural deficiencies, considering the thoroughness of this approach.

## 2. Methods

### 2.1. Cell Culture

**Figure 1:**
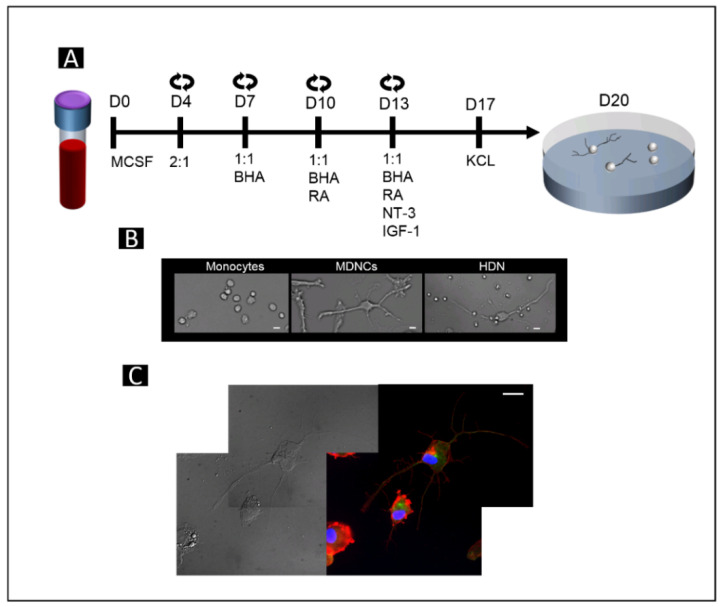
Transdifferentiation of human circulating monocytes into neuronal-like cells. (**A**) Schematic representation of our 20-day protocol for transdifferentiating human circulating monocytes into neuronal-like cells, starting from day zero (D0) with a blood sample and ending on D20 with neuronal-like cells. Circled arrows represent days on which media were changed. Cell cultured media were replaced with new DMEM, together with PBMCs conditioned media, at a rate of either 2:1 or 1:1 (DMEM/PBMCs), depending on the day of culture, as depicted in the diagram. DMEM was supplemented with different chemicals and growth factors depending on the day of culture, as depicted in the diagram. Exact concentrations are described in the Materials and Methods section. (**B**) Light microscopy photographs of monocytes, right after isolation from PBMCs, monocyte-derived-neuronal-like cells (MDNCs) and human developing neurons (HDNs) in culture for 5 days (20× original magnification). (**C**) Light microscopy photographs of MDNCs in parallel with immunostainings showing tubulin in green and actin in red. The cells’ nuclei were stained with DAPI in blue (60× original magnification). Scale bar = 20 µm.

All blood donors gave their informed and written consent after receiving a full description of the study. Experiments were approved by the Institutional Review Board (IRB) at Penn State University (Study #00006911). Fresh blood was obtained from healthy individuals. We then followed our transdifferentiation protocol, as previously described [28]. Briefly, fresh blood was separated into its components by Ficoll-Paque (17-1440-03, GE Healthcare, Chicago, IL, USA). A fraction of peripheral blood mononuclear cells (PBMCs) was cultured on fibronectin-coated 25 cm^2^ flasks (13.5 million PBMCs per flask). The remaining PBMCs were used for isolation of CD14+ cells (monocytes) by positive immunomagnetic selection based on the manufacturer protocol (CD14 human microbeads, 130-050-201 Miltenyi Biotec, Auburn, CA, USA). CD14+ cells were cultured on fibronectin-coated wells at a concentration of 180,000 cells per cm^2^. Plastic plates and flasks came from BD Falcon, Glendale, AZ, USA (351146, 353043 and 353109). Human fibronectin from plasma (F2006, Sigma-Aldrich, St. Louis, MO, USA) was used at a concentration of 20 µg/mL, and coating was carried out overnight at 4 °C. Macrophage colony-stimulating factor (MCSF) from AbCys, Paris, France (300-25) was added to monocytes right before culturing at a final concentration of 50 ng/mL (Figure 1A). All cells were maintained in Dulbecco’s modified Eagle medium (DMEM), high glucose, GlutaMAX (61965059, GIBCO, Waltham, MA, USA), in which we added 100 U/mL penicillin; 100 mg/mL streptomycin, 1% nonessential amino acids, 1 mM sodium pyruvate, 10 mM HEPES buffer, (all from Life Technologies, Waltham, MA, USA) and supplemented with 10% fetal bovine serum (FBS) Performance Plus from GIBCO (Waltham, MA, USA). Cell culture medium was then replaced on days 4, 7, 10 and 13, as described in Figure 1. The following chemicals and growth factors were added, as shown in Figure 1: butylated hydroxyanisole (BHA) (B1253, Sigma-Aldrich, St. Louis, MO, USA), retinoic acid (RA) (R2625, Sigma-Aldrich, St. Louis, MO, USA), insulin growth factor-1 (IGF-1) (100-11, PeproTech, Cranbury, NJ, USA) and neurotrophin-3 (NT-3) (450-03-100, Peprotech, Cranbury, NJ, USA). On day 17, cell culture media was not replaced; instead, 25 mM potassium chloride (KCL) was added (P5405, Sigma-Aldrich, St. Louis, MO, USA).

Pictures of cells were taken using a Nikon (Melville, NY, USA) Eclipse Ti-S/L 100 inverted microscope equipped with a CoolSNAP Myo, 20 MHz, 2.8 Megapixel, 4.54 × 4.54 µm pixels camera (Melville, NY, USA) and with a Nikon CFI Super flour 20X DIC prism objective (Melville, NY, USA). Pictures were taken immediately after monocyte extraction and on days 20–21 when transdifferentiation was completed (Figure 1B). Pictures of transdifferentiated cells either under control conditions or after treatment with colchicine 0.5 µM (Sigma-Aldrich, C9754) were identified via a micro-ruled coverslip (Cellattice CLS5-25D, Nexcelom Bioscience, Lawrence, MA, USA). Only neuronal-like cells with at least one primary neurite longer than 2 times the soma size before treatment were traced.

Immunofluorescence was performed as previously described [28] (f. Briefly, after fixation and permeabilization, cells were stained with 4′,6-diamidino-2-phenylindole, dihydrochloride (DAPI, D1306, Thermo Fisher Scientific, Waltham, MA, USA), mouse anti-tubulin (1/100, Invitrogen, Waltham, MA, USA), Alexa Fluor-488 (1/200, Life Technology, Waltham, MA, USA) and rhodamine phalloidin (1/200, Invitrogen, Waltham, MA, USA). Images were visualized with a Leica DMI 6000 microscope (Wetzlar, Germany) equipped with a Micro MAX-1300YHS camera using an HCX PL APO 60X oil objective (Leica, Wetzlar, Germany). Images were captured using Metamorph Software (Version 7.1.3, Molecular Devices, San Jose, CA, USA).

### 2.2. Single Cell RNA-Sequencing

We utilized microfluidic single-cell capture and single-cell mRNA sequencing technologies via Fluidigm’s C1^TM^ Single-Cell Autoprep System (C1) to explore genome-wide gene expression in 17 cells exposed to our transdifferentiation protocol and for THP-1 cells. We followed the manufacturer’s protocol, as previously described [28]. In short, cells were loaded using an integrated fluidic circuit (IFC) chip that allowed capturing a single cell per well. After optical confirmation of cell number at each capture site on the chip, the cells were processed for in-line cell lysis, reverse transcription and cDNA amplification steps. The resulting cDNA was converted to a sequencing library using Illumina’s Nextera XT library preparation kit. The *Rapid* mode of Illumina HiSeq 2500 was used to generate sequencing reads of sufficient depth (about 3 million of sequencing reads) per each cell. De-multiplexed sequencing reads passed the default quality filtering of the Illumina CASAVA pipeline (v1.8, Ilumina, Inc., San Diego CA, USA) and were then exposed to further quality trimming/filtering using FASTX-Toolkit (v.0.0.13, Hannon Laboratory, Cold Spring Harbor, NY, USA). The filtered reads were aligned to the most recent reference genome (hg38) using Tophat (v2.0.9, Center for Computational Biology, Baltimore, MD, USA) [36] by allowing up to 2 mismatches. After normalization was performed via the median of the geometric means of fragment counts across all libraries, fragments per kilobase per million (FPKM) mapped reads values were calculated using Cuffdiff tool, which is available in Cufflinks version 2.2.1 (Trapnell Lab, Seattle, WA, USA) [37]. Some results from this experiment were reported previously [28], but the expression of all genes presented in this manuscript have never before been reported in MDNCs.

Gene expression for human neuroblastoma cells was obtained from a public database generated by Li et al. [38].

### 2.3. Statistical Analysis

The non-parametric Mann–Whitney test was used to make pairwise comparisons between MDNCs treated with colchicine versus MDNCs under control conditions. A one-way ANOVA followed by Bonferroni correction was used to make comparisons between the time it took to trace MDNCs using each of the three tracing methods tested. *p* values lower or equal to 0.05 were considered significant. 

## 3. Results

### 3.1. Neuronal and Monocyte Markers in MDNCs, SH-SY5Y and THP-1 Cells

We compared the expression of 12 neuronal markers between (a) MDNCs; (b) SH-SY5Y cells, a human neuroblastoma cell line commonly used to study neuronal processes; and (c) THP-1 cells, a human monocytic cell line (to serve as negative control). Of the 12 neuronal markers, 7 are involved in synaptic functions, 4 are part of the neuronal structure and one is a gamma-aminobutyric acid (GABA) type A receptor (Table 1). The expression of these 12 genes has never been reported in MDNCs. Expression of these 12 genes in 17 MDNCs was determined by single-cell mRNA sequencing. All 12 neuronal markers were expressed in at least one MDNC, and most genes were expressed in at least 6 MDNCs (Table 2). SH-SY5Y cells also expressed all of these neuronal genes, while they were not expressed in THP-1 cells (Table 2). We then tested whether two markers for monocytes were present in THP-1 cells, MDNCs or SH-SY5Y. As expected, these two monocyte-specific genes were highly expressed by THP-1 cells, whereas they were not expressed by undifferentiated neuroblastoma cells and were barely detectable in differentiated SH-SY5Y cells (Table 2). Only 1 out of the 17 MDNCs showed very low expression of 1 monocyte marker, and none were expressed in the remaining 16 MDNCs (Table 2).

### 3.2. Whole-Cell Tracing

**Figure 2:**
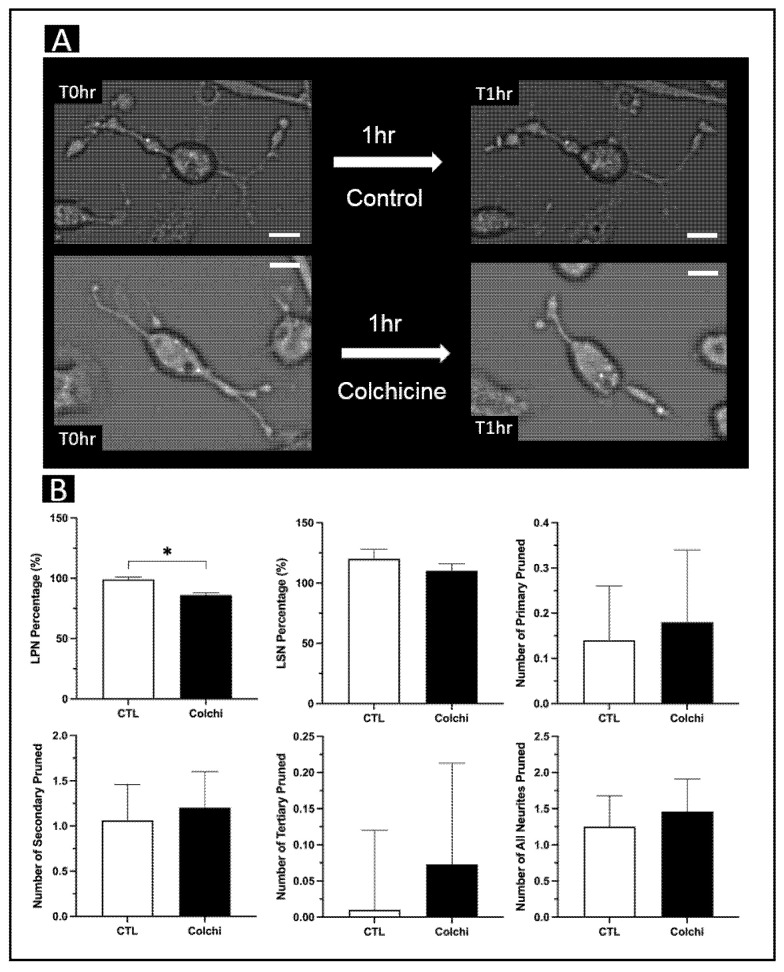
Whole-cell tracing of MDNCs after treatment with colchicine 0.5 µM. (**A**) Light microscopy photographs of the exact same MDNCs before (T0 h) and after one hour (T1 h) under control conditions or after treatment with colchicine 0.5 µM (20× original magnification). Scale bar = 20 µm. (**B**) Bar graphs comparing MDNCs’ structural response to colchicine versus MDNCs under control conditions. Structural parameters include longest primary neurite (LPN), longest secondary neurite (LSN), number of primary neurites, number of secondary neurites, number of tertiary neurites and total number of neurites. Data are presented as mean ± SEM. Differences were assessed using the non-parametric Mann–Whitney test. For LPN, number of primary, number of secondary, number of tertiary and total number of neurites, *n* = 96 for control and *n* = 82 for colchicine. For LSN, *n* = 91 for control and *n* = 75 for colchicine. * *p* = or < 0.05.

After 20 days in culture following our protocol [28], transdifferentiated monocytes acquired a neuronal morphology comparable with that of HDNs (Figure 1B). These MDNCs extended neurites with a microtubule-based shaft, as shown in Figure 1C. Colchicine is well-known for its ability to elicit neurite retraction [53,54] via microtubule depolymerization [55]. In a prior publication, we showed that the structure of MDNCs responds similarly to the structure of neuroblastoma cells and that of human neurons in vitro when treated with colchicine 0.5 µM [28]. While retraction is expected with colchicine 0.5 µM, minimal to no retraction should occur under control conditions. To determine whether MDNCs exhibited any retraction under control culture conditions, a group of MDNCs was identified and photographed at baseline, meaning at time zero (T0 h). These MDNCs were kept under control conditions for 1 h (T1 h), and pictures of the exact same MDNCs were taken again (Figure 2A). The same procedure was followed to establish whether colchicine elicited pruning of neuronal extensions. In this latter case the 1 h incubation period was carried out in the presence of colchicine 0.5 µM (Figure 2A).

The principal investigator (PI), who has ample experience tracing cells, traced these four sets of MDNCs, meaning cells that were cultured under control conditions at T0 h and T1 h, as well as cells treated with colchicine at T0 h and T1 h. Since the entire neuropil of each MDNC was traced, we can report differences in the longest primary neurite (LPN), longest secondary neurite (LSN), number of primary neurites, number of secondary neurites, number of tertiary neurites and total number of neurites. MDNCs were traced using a semi-automated software called FIJI (NIH, Bethesda, MD, USA), which is a plugin for ImageJ, an open source image processing program provided by the National Institutes of Health (NIH). 

To determine whether there was retraction of LPN or LSN, the percentage of neurite remaining at T1 h was calculated (T1 h/T0 h) for MDNCs cultured under control conditions, as well as cells treated with colchicine 0.5 µM. Then, non-parametric statistical analyses were conducted to establish whether there were differences between control (CTL) and colchicine (Colchi). Whole-cell tracing by the PI evidenced a statistically significant reduction in the percentage of LPN after treatment with colchicine 0.5 µM (CTL, 99 ± 2%; Colchi, 86 ± 2%; *p* = 0.0006), while there were no differences in LSN (CTL, 120 ± 8%; Colchi, 110 ± 6%; *p* = 0.22) (Figure 2B). To establish differences in the number of neurites pruned, we subtracted the number of neurites at T0 h from the number of neurites at T1 h for MDNCs cultured under control conditions as well as for cells treated with colchicine 0.5 µM. Whole-cell tracing did not reveal differences in the number of primary (CTL, 0.14 ± 0.12; Colchi, 0.18 ± 0.16; non-parametric analysis *p* = 0.48), secondary (CTL, 1.06 ± 0.4; Colchi, 1.2 ± 0.4; *p* = 0.45), tertiary (CTL, 0.010 ± 0.11; Colchi, 0.073 ± 0.14; *p* = 0.52) or total neurites pruned (CTL, 1.25 ± 0.43; Colchi, 1.46 ± 0.45; *p* = 0.27) (Figure 2B).

### 3.3. Three Neurite Tracing Approaches

Three neurite tracing approaches were tested by four individuals with research experience (two medical students with previous research experience, one neuroscience graduate student and one laboratory technician). One of the medical students had performed cell tracing before participating in this study, whereas all other participants had no experience in tracing. The tracers were blinded to the treatment condition each of the two groups of MDNCs had received (either CTL or Colchi), and they were unaware of that we were expecting pruning of neuronal extensions with Colchi. Participants were trained on how to use FIJI (NIH, Bethesda, MD, USA), a semi-automated software, and Volocity (Quorum Technologies, Ontario, Canada), an automated software. After training was completed, participants were told to use FIJI for whole-cell tracing, and they were instructed to trace all neurites present in MDNCs for this first tracing method. For the second tracing method, they were again instructed to use FIJI, but this time to trace only the longest primary and longest secondary neurite in each of the MDNCs. This second approach was named the longest neurite method (LN). For the third method, participants processed the photographs of MDNCs for each of the two treatment conditions through Volocity and then confirmed that the MDNCs had been traced. Finally, participants were asked to record the time it took them to trace MDNCs with each of the three tracing methods.

**Figure 3:**
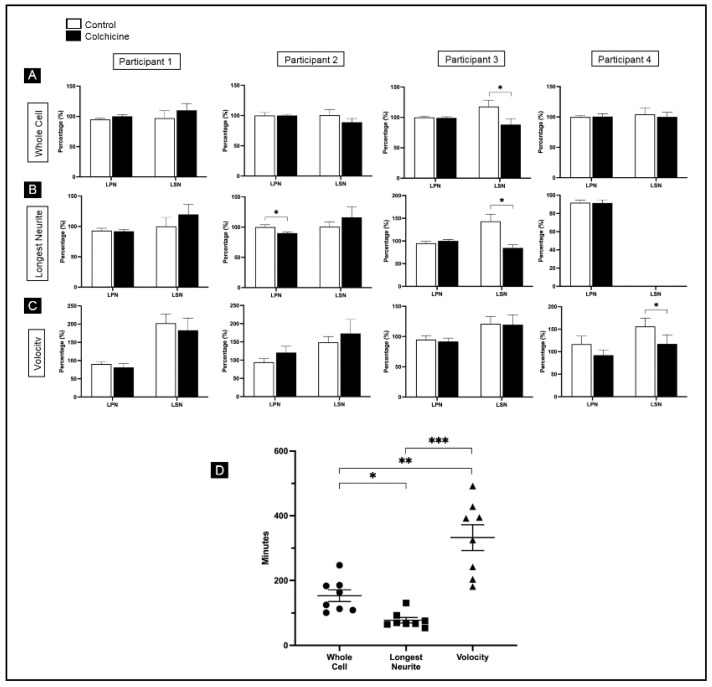
Comparison between three different tracing methods. (**A**) Bar graphs comparing MDNCs’ structural response to colchicine versus MDNCs under control conditions after tracing each MDNC in its entire. Structural parameters include longest primary neurite (LPN) and longest secondary neurite (LSN). Data are presented as mean ± SEM. Differences were assessed using the non-parametric Mann–Whitney test. Participant 1 (P1): for LPN *n* = 37 for control and *n* = 32 for colchicine; for LSN, *n* = 23 for control and *n* = 20 for colchicine. P2: for LPN *n* = 38 for control and *n* = 35 for colchicine; for LSN, *n* = 15 for control and *n* = 19 for colchicine. P3: for LPN *n* = 47 for control and *n* = 34 for colchicine; for LSN, *n* = 41 for control and *n* = 29 for colchicine. P4: for LPN *n* = 51 for control and *n* = 30 for colchicine; for LSN, *n* = 31 for control and *n* = 15 for colchicine. (**B**) Bar graphs comparing MDNCs’ structural response to colchicine versus MDNCs under control conditions after only tracing LPN and LSN. The statistical assessment and data presentation are the same as in (**A**). P1: for LPN *n* = 39 for control and *n* = 36 for colchicine; for LSN, *n* = 22 for control and *n* = 17 for colchicine. P2: for LPN *n* = 38 for control and *n* = 36 for colchicine; for LSN, *n* = 21 for control and *n* = 17 for colchicine. P3: for LPN *n* = 30 for control and *n* = 35 for colchicine; for LSN, *n* = 28 for control and *n* = 34 for colchicine. P4: for LPN *n* = 42 for control and *n* = 37 for colchicine; P4 did not trace LSN. (**C**) Bar graphs comparing MDNCs’ structural response to colchicine versus MDNCs under control conditions after tracing MDNCs’ length and width using the automated tracing software Volocity. The statistical assessment and data presentation are the same as in (**A**). P1: for LPN *n* = 30 for control and *n* = 26 for colchicine; for LSN, *n* = 30 for control and *n* = 23 for colchicine. P2: for LPN *n* = 25 for control and *n* = 18 for colchicine; for LSN, *n* = 25 for control and *n* = 18 for colchicine. P3: for LPN *n* = 33 for control and *n* = 30 for colchicine; for LSN, *n* = 33 for control and *n* = 30 for colchicine. P4: for LPN *n* = 27 for control and *n* = 24 for colchicine; for LSN, *n* = 27 for control and *n* = 24 for colchicine. * *p* = or < 0.05. (**D**) Dot plot comparing the time in minutes necessary for completing the tracing of all MDNCs (control + colchicine) with each of the three tracing methods: whole cell, longest neurite and Volocity. Data are presented as mean ± SEM. Differences were assessed using a one-way ANOVA followed by Bonferroni correction. For whole cell, longest neurite and Volocity *n* = 8. * *p* < 0.005, ** *p* < 0.002 and *** *p* < 0.00002.

Volocity automatically traces the length and width of the cell, which in the case of neuronal-like cells approximates to the longest primary neurite and the longest secondary neurite. Since two of the three tracing methods tested only provided information on LPN and LSN, we compared the three tracing approaches based on these two neuronal extensions. None of the four individuals encountered any statistically significant difference in LPN after whole-cell tracing (participant 1 (P1), CTL, 95 ± 2%; Colchi, 100 ± 3%; *p* = 0.35; P2, CTL, 100 ± 5%; Colchi, 100 ± 2%; *p* = 0.86; P3, CTL, 100 ± 2%; Colchi, 99 ± 2%, *p* = 0.44; P4, CTL, 99.9 ± 2.8%; Colchi, 100 ± 4.6%; *p* = 0.45) (Figure 3A). Participant 3, however, found colchicine elicited a statistically significant retraction of LSN (P3, CTL, 118 ± 10.3%; Colchi, 88.1 ± 9.7%; *p* = 0.004), while all other participants observed no differences (P1, CTL, 97 ± 12.4%; Colchi, 109.9 ± 11.3%; *p* = 0.24; P2, CTL, 100.6 ± 9.5%; Colchi, 88.9 ± 6.2%; *p* = 0.27; P4, CTL, 104.5 ± 10%; Colchi, 100.1 ± 7.5%; *p* = 0.71) (Figure 3A).

When tracing only longest neurites, one of the participants found colchicine elicited a statistically significant retraction of LPN when compared with MDNCs under control conditions (P2, CTL, 100 ± 4%; Colchi, 90 ± 2%; *p* = 0.04) (Figure 3B). All other participants found no statistical differences in LPN (P1, CTL, 93 ± 4%; Colchi, 92 ± 3%; *p* = 0.66; P3, CTL, 95 ± 4%; Colchi, 100 ± 3%; *p* = 0.34; P4, CTL, 91.7 ± 2.7%; Colchi, 91.4 ± 3.4%; *p* = 0.42). The same participant who found a significant retraction of LSN while tracing the entire cell again encountered retraction elicited by colchicine while tracing only longest neurites (P3, CTL, 143.2 ± 15.8%; Colchi, 84.6 ± 7%; *p* = 0.001) (Figure 3B). Two other participants found no statistical differences in LSN (P1, CTL, 100 ± 14.9%; Colchi, 119.7 ± 16.3%; *p* = 0.07; P2, CTL, 100.7 ± 7.8%; Colchi, 116.2 ± 17.1%; *p* = 1.0), and one participant did not trace LSN (Figure 3B).

The use of Volocity rendered no statistically significant differences in LPN for any of the participants (P1, CTL, 90.2 ± 5.5%; Colchi, 81.4 ± 11%; *p* = 0.14; P2, CTL, 94.6 ± 9.6%; Colchi, 120.7 ± 17.4%; *p* = 0.18; P3, CTL, 95 ± 6.4%; Colchi, 91.7 ± 5.7%, *p* = 0.68; P4, CTL, 116.6 ± 18.2%; Colchi, 92.2 ± 11.8%; *p* = 0.27) (Figure 3C). However, participant 4 found a significant retraction in LSN after treatment with colchicine (P4, CTL, 156.2 ± 18.4%; Colchi, 117 ± 20.3%; *p* = 0.05) (Figure 3C). None of the other participants found statistical differences in LSN while using Volocity (P1, CTL, 202.5 ± 25.5%; Colchi, 182.9 ± 33.6%; *p* = 0.27; P2, CTL, 148.6 ± 16.1%; Colchi, 173 ± 38.5%; *p* = 0.99; P3, CTL, 121.2 ± 12%; Colchi, 119.4 ± 16.4%; *p* = 0.31) (Figure 3C).

A one-way ANOVA revealed that the amount of time necessary to complete all tracings was significantly different between each of the three approaches (F(2, 21) = 25.74, *p* < 0.00001) (Figure 3D). Bonferroni correction indicated that tracing longest neurites (LN) took less than half of the time needed to trace the whole cell (WC) (LN, 77.8 ± 8.5 min; WC, 153.6 ± 17.9 min; *p* = 0.001), while tracing the entire cell was more efficient than using Volocity (WC, 153.6 ± 17.9 min; V, 332.5 ± 39.9 min; *p* = 0.001) (Figure 3D). Since one of the students did not trace longest secondary neurites when applying the LN approach, we ran another one-way ANOVA excluding that individual’s LN data. The results remained significant (F(2, 19) = 20.81, *p* = 0.00001).

## 4. Discussion

We have previously shown that MDNCs conduct electrical activity and express a wide variety of neuronal markers [28]. Here we expanded the list to include 12 neuronal genes: 7 involved in synaptic transmission [39,40,41,42,43,44,45], 4 associated with neuronal structure [46,47,48,49] and 1 gamma-aminobutyric acid (GABA) receptor [50] (Table 1 and Table 2). Several of these genes are implicated in the pathophysiology of neurodevelopmental illnesses. For instance, neurexin 3 has been linked to autism [56], whereas SV2A and VAMP are associated with schizophrenia [57,58]. Another synaptic gene, SNAP-25, has been implicated in the etiology of both illnesses [59,60]. Tau and GAP-43 are essential for the development of neuronal structure [47,49]. While tau is commonly known for its association with Alzheimer’s disease, this protein has also been linked to schizophrenia [61]. Similar to tau, GAP-43 is crucial for outgrowth of neuronal extensions [49], and not surprisingly, abnormalities in the expression of GAP-43 have been associated with both schizophrenia [62] and autism [63]. Other proteins relevant for the establishment of neuronal shape during development and often involved in the pathophysiology of schizophrenia, such as MAP-2 [64,65], are also expressed by MDNCs [28]. At the same time, markers for monocytes such as CD11B and CCR2 [51,52] are no longer present in MDNCs (Table 1 and Table 2).

Several lines of evidence strongly indicate that deficits in the neuronal structure are implicated in the pathophysiology of autism and schizophrenia [12,13,66,67,68]. However, the inaccessibility of neurons coming directly from living patients’ brains has limited the study of early neurodevelopmental processes that transform neuronal structure. MDNCs not only express a variety of genes crucial in sculpting neuronal shape, but in addition, the structure of MDNCs is comparable with that of human neurons after 5 days in culture and also with that of differentiated human neuroblastoma cells [28]. Moreover, the structure of MDNCs responds similarly to that of neurons and neuroblastoma cells when treated with dopamine and colchicine [28]. 

MDNCs’ ability to reproduce characteristics of the structure of human neurons opens the opportunity for studying these aspects of neurodevelopmental illnesses directly in living patients’ cells. This means that MDNCs provide a window into early neurodevelopmental processes in vitro, even when patients are already adults. Nonetheless, in order to maximize the delivery of neurostructural results, it is imperative to determine which neurite tracing method is more efficient in extracting data from MDNCs.

Unfortunately, there is no universal tracing method that can efficiently extract neurostructural data under all research conditions. Instead, experts recommend testing several tracing approaches to determine which is the best suited for each laboratory [69,70]. Currently, there is a plethora of automated tracing methods, but the gold standard continues to be manual tracing via semi-automated approaches [69]. Therefore, here we tested three different tracing approaches: (1) whole-cell tracing, (2) longest neurite tracing and (3) Volocity. The first two are semi-automated and thus require more work, while the third method is completely automated. However, before comparing these three tracing methods, we had to establish the right conditions for comparison. Therefore, the principal investigator, who has ample experience tracing cells, traced two separate groups of MDNCs: one cultured under control conditions and one treated with colchicine 0.5 µM. This compound is well-known for its capacity to cause neurite retraction via microtubules depolymerization [55]. Furthermore, we have previously shown that colchicine elicits pruning of neuronal extensions in MDNCs in a way similar to what is found in neurons [53] and neuroblastoma cells [54]. 

Using the more thorough tracing approach—namely, whole-cell tracing—the PI found that colchicine elicited, as expected, a statistically significant retraction of LPN (Figure 2B). None of the other structural parameters revealed statistical differences (Figure 2B). Then, four other individuals, mostly students with research backgrounds, traced the same two groups of MDNCs (control versus colchicine 0.5 µM) using the three different tracing methods. These four participants were blinded to the treatment condition they were tracing. All the statistically significant retractions found by these four participants were, as expected, caused by colchicine (Figure 3A–C). However, relatively few structural differences were found. This is not entirely surprising, as these four individuals had limited experience with tracing, and the differences between the two MDNCs groups were subtle (Figure 2B). It is important to note that the tracing approach that yielded more statistically significant findings was the simplest of all, meaning the approach that only traced the longest primary and longest secondary neurite (Figure 3B). 

The most surprising finding was that Volocity, the automated tracing method, was the slowest in delivering structural results (Figure 3D). This delay was not due to lack of recognition of MDNCs, even though these cells were not marked with a fluorochrome. Instead, pictures of MDNCs were live. The difficulties arose because in many instances Volocity did not identify the entire neuritic length. Students, therefore, had to piece together sections of neurites, similar to what other research teams have described using different automated softwares [70]. This task was more time consuming than even tracing the entire cell using a semi-automated approach (Figure 3D). Having MDNCs stained with a fluorochrome would have eliminated the need for reconstruction of the neuritic length. However, given the inherent damage attached to cell fixation and permeabilization [34,35], it is questionable whether the subtle structural differences between control MDNCs and those treated with colchicine would have been observed. 

The fastest tracing approach was tracing only the longest primary and longest secondary neurites (Figure 3D). This approach was also the one that yielded more statistically significant differences between treatment conditions (control versus colchicine 0.5 µM) (Figure 3B). We were expecting whole-cell tracing to detect more structural differences, given the precision of this method. However, perhaps the simplicity of tracing only two neurites per MDNC as opposed to delineating the entire neuropil improves accuracy. Given that tracing longest neurites was more accurate and took half the time as tracing the entire cell and a quarter of the time as Volocity (Figure 3D), we recommend this tracing approach for future studies on the structure of MDNCs.

There are other factors that need to be considered when selecting a tracing approach. One is that tracing only the longest neurites neglects other structural parameters. Additionally, Volocity is just one of the many commercially available automated software products. It is possible that other automated applications would recognize the entire structure of MDNCs, even when analyzing photographs of live cells using light microscopy. However, if automated software products become a viable alternative, cost will have to be factored in, as semi-automated methods usually do not bear any cost to the researcher, while most automated software have to be purchased [69,70]. 

In summary, selecting a tracing method is a complicated process that depends on the specific research conditions to be tested [69,70]. For instance, analyzing neurons in culture (2-dimensional) versus brain slices (3-dimensional) or studying intact neurites versus damaged neurites would each generate its own set of intricacies for which only a couple of tracing approaches would be suitable. It is also essential to determine which aspects of the neuronal structure will be studied, as some tracing paradigms are better at measuring neurite length, while others excel at counting number of extensions [69,70]. Therefore, experts recommend testing different tracing approaches to determine the most efficient method for each laboratory [69,70]. Here, we determined that the most efficient tracing strategy for studying neuritic length in MDNCs is tracing only the longest primary and longest secondary neurites (Figure 3D). The limitation of this modality is that it does not provide information about the number of neurites or other aspects of the neuropil, such as number or length of tertiary or quaternary neurites. Another limitation of our study is that we only conducted tracing using one automated method among the many currently available [69,70]. Future studies will have to be conducted to determine whether other automated paradigms prove better at extracting neurostructural data from MDNCs. 

## 5. Conclusions

MDNCs express a wide variety of neuronal markers that have been associated with the pathophysiology of autism and schizophrenia. Since MDNCs originate from a blood sample taken directly from patients, these cells carry the genetic susceptibility to the neurodevelopmental illness that the patients are afflicted with. In contrast with rodent neurons in culture or neuronal cell lines such as neuroblastoma cells, MDNCs allow researchers to study directly in patients’ cells early neurodevelopmental processes involving changes in neuronal structure. In order to maximize efficiency in studying MDNCs’ structure, the best approach is to only trace the longest primary neurite and the longest secondary neurite using FIJI, a semi-automated software made available by the NIH.

## Figures and Tables

**Table 1:** Neuronal and monocytic genes and their functions.

Protein	Gene	Identifier	Function	Reference
Neurexin 3	NRXN3	ENSG00000021645	Synapsis	Sudhof 2021 [39]
Synaptosome-associated protein 25	SNAP25	ENSG00000132639	Synapsis	Antonucci et al. 2016 [40]
Synaptic vesicle glycoprotein 2A	SV2A	ENSG00000159164	Synapsis	Nowack et al. 2010 [41]
Vesicle-associated membrane protein 1	VAMP1	ENSG00000139190	Synapsis	Bhattacharya et al. 2002 [42]
SH3 and multiple ankyrin repeat domains 2	SHANK2	ENSG00000162105	Synapsis	Lim et al. 1999 [43]
Synuclein alpha	SNCA	ENSG00000145335	Synapsis	Burre 2015 [44]
Syntaxin 1A	STX1A	ENSG00000106089	Synapsis	Bennett et al. 1992 [45]
Spire type actin nucleation factor 1	SPIRE1	ENSG00000134278	Neuronal structure	Schumacher et al, 2004 [46]
Microtubule-associated protein tau	MAPT	ENSG00000186868	Neuronal structure	Barbier et al. 2019 [47]
Shootin 1	SHTN1 (KIAA1598)	ENSG00000187164	Neuronal structure	Toriyama et al. 2006 [48]
Growth-associated protein 43	GAP43	ENSG00000172020	Neuronal structure	Meiri et al. 1986 [49]
Gamma-aminobutyric acid (GABA) type A receptor subunit beta 3	GABRB3	ENSG00000166206	GABA receptor	Mortensen et al. 2010 [50]
Integrin subunit alpha M CD11B	ITGAM	ENSG00000169896	Immune system	Schmid et al. 2018 [51]
Monocyte chemoattractant protein 1 peceptor	CCR2	ENSG00000121807	Immune system	Tu et al. 2020 [52]

**Table 2:** Expression of 12 neuronal markers and 2 markers for monocytes in THP-1 monocytic cells, SH-SY5Y neuroblastoma cells and 17 MDNCs.

Gene	THP-1	1	2	3	4	5	6	7	8	9	10	11	12	13	14	15	16	17	SH-SY5Y 1 *	SH-SY5Y 2 *
NRXN3	0	0	0	0.101	3.106	0.04	0.012	0	0.009	0	0.288	0	0	0.062	0.061	0.022	0	0.98	0.01	0.135
SNAP25	0	0	0	0.779	0.167	0.13	0.219	0.401	0	0	0	0	1.19	0.288	0	0	0.171	0	25.06	42.23
SV2A	0	0	0	0.089	0	0	0	0	0	0.127	0.044	0	0	0	0.086	0.171	0	0.055	10.37	6.810
VAMP1	0	0	0	0	0	0.256	0	0	0	0	0.692	0	0	0	0.054	0	0	0	0.942	1.472
SHANK2	0	0	0	0	0	0.132	0.031	0	0.76	0.068	0.929	0	0.059	0	0.223	0.552	0	0.199	0.219	0.229
SNCA	0	5.35	8.08	0	0	0.811	1.12	0	0	0	0	0	276.9	0	0	136.3	1.63	0	3.776	7.513
STX1A	0	0	0	0	0	0	0.028	0	0	0	0	0	0	0	0	0	0	0	8.742	9.759
SPIRE1	0	27.2	58.02	0.292	5.19	0	0.01	0	16.34	0.223	3.37	0	0	17.32	28.61	110.5	2.85	23.25	9.341	6.986
MAPT	0	0.089	0.023	0	0	0.075	0.192	0.024	0.128	0	0.045	0.042	0.028	0	0	0.019	0.032	0.073	1.535	4.176
SHTN1	0	45.73	25.8	4.66	0.27	4.62	2.47	43.59	4.53	16.44	20.04	0	0	1.36	12.95	0.154	0	83.34	2.038	3.827
GAP43	0	0	0	0	0.211	0	0	0	0	0	0	0	0	0	0	0	0	0	62.16	86.84
GABRB3	0	0.324	0.458	0.065	0.254	0.247	0.079	0.095	0.21	0.13	0.06	0.225	0	1.06	0.093	0.901	0.173	0.182	10.25	9.763
ITGAM	14.91	0	0	0	0	0	0	0	0	0	0	0	0.034	0	0	0	0	0	0	0.014
CCR2	24.62	0	0	0	0	0	0	0	0	0	0	0	0	0	0	0	0	0	0	0.078

1 * undifferentiated SH-SY5Y; 2 * differentiated SH-SY5Y; data from Li et al. 2015 [38].

## Data Availability

The data presented in this study are available on request from the corresponding author.
